# Female Sex and Obesity Are Risk Factors for Inadequate Calcium Intake in Youth With Type 1 Diabetes

**DOI:** 10.3389/fcdhc.2021.723855

**Published:** 2021-09-06

**Authors:** Roman Rahmani, Elizabeth Stevens, Noya Rackovsky, Kimberly O. O’Brien, George J. Schwartz, David R. Weber

**Affiliations:** 1 Department of Pediatrics, Endocrinology, Golisano Children’s Hospital, University of Rochester Medical Center, Rochester, NY, United States; 2 Division of Nutritional Sciences, Cornell University, Ithaca, NY, United States

**Keywords:** type 1 diabetes, calcium, obesity, bone health, fracture

## Abstract

People with type 1 diabetes (T1D) are at increased risk of developing low bone mineral density and fractures. Optimization of calcium intake is a key component of pediatric bone health care. Despite the known risk factors for impaired bone health in T1D and the known benefits of calcium on bone accrual, there are limited data describing calcium intake in youth with T1D. In this cross-sectional study, calcium intake was assessed in 238 youth with T1D. One third of study participants were found to have inadequate calcium intake. Female sex, especially during adolescence, and obesity were identified as specific risk factors for inadequate calcium intake. Given the known adverse effects of T1D on bone health, efforts to promote calcium intake in youth with T1D should be considered.

## INTRODUCTION

Type 1 diabetes (T1D) is a chronic autoimmune disease characterized by destruction of the pancreatic beta cells, absolute insulin deficiency and hyperglycemia. The condition is treatable, but requires lifelong daily administration of multiple doses of insulin. In addition to acute complications related to hyper- and hypoglycemia, patients with T1D are at risk of developing end organ damage including retinopathy, nephropathy, and neuropathy. More recently, impaired bone health has emerged as an additional complication of T1D (1). The majority of patients living with T1D develop the disease during childhood prior to the attainment of peak bone mass (2). Multiple studies have shown that the growing skeleton is adversely affected by T1D. Children with T1D have been found to have an increased risk of fracture (3), deficits in bone density (4) and altered bone microarchitecture (5) compared to healthy peers.

The pathophysiology underlying skeletal damage in T1D remains poorly understood and is likely multi-factorial. One factor hypothesized to contribute to impaired bone health in T1D is altered calcium metabolism. In particular, urinary calcium excretion has been shown to be elevated in T1D youth (6) and may affect calcium availability for bone mineralization. Dietary calcium intake is a potentially modifiable factor affecting calcium availability for bone accrual and has been associated with higher bone density in children (7). There are limited data characterizing calcium intake in youth with T1D. Given the lifelong increased fracture risk in people with T1D, it is imperative that factors affecting bone accrual, including calcium intake, be maximized during the proliferative adolescent years of bone mineralization. The objective of this study was to assess dietary and supplemental calcium intake in a clinical sample of youth with T1D and to identify risk factors for insufficient calcium intake.

## METHODS

Patients between the ages of 5-21 years with a T1D diagnosis who received care at the University of Rochester (URMC) Pediatric Endocrinology Clinic between 2016-2017 were eligible to participate in this cross-sectional study. Additional inclusion criteria included duration of T1D of at least 1 year, and BMI <99^th^ percentile for age and sex (to reduce likelihood of including subjects with type 2 diabetes). Non-English speaking/reading patients were excluded. Participants were recruited in-person by a member of the study team *via* convenience sampling of consecutive patient visits on clinic days.

The Short Calcium Questionnaire, revised for pediatric use by bionutritionists at URMC, was used to assess calcium intake. This previously validated food frequency questionnaire (FFQ) was designed to rapidly assess usual calcium intake based upon the number of food and supplement servings consumed in a typical week (8). The FFQ was administered to subjects (with parent/guardian assistance, as needed) in-person by a trained study-team member. Participants were selected by convenience sampling of patients at the time of regularly scheduled clinical visits. The recommended dietary allowance (RDA) as determined by the Institute of Medicine was used to assess adequacy of calcium intake (9). The RDA for calcium represents the calcium intake anticipated to meet the requirements of at least 97.5% of individuals and varies by age for the study population as follows: 5-8 years, 1000 mg/day; 9-18 years, 1300 mg/day; 20-21 years, 1000 mg/day. RDA was selected as the primary study outcome in this study over the estimated average requirement (EAR, calcium intake corresponding to the median requirement of the population) because 1) the RDA is more commonly used for clinical care (including at URMC), 2) has been more commonly reported in studies of T1D, and 3) it was viewed by the investigators to be a more clinically appropriate outcome in a population known to be at risk for impaired bone accrual.

Glycemic status was assessed by hemoglobin A1c (HbA1c) on day of the study visit and by average HbA1c from diagnosis (obtained by medical chart review). Height was obtained by wall mounted stadiometer and weight by digital stand on scale. BMI was calculated as weight(kg)/height(m)^2^. Obesity was defined as BMI ≥ 95^th^ percentile for age and sex using national reference data (10). Subject demographics and medical history were assessed by questionnaire and chart review. A subset of subjects secondarily had dietary calcium intake assessed by 24-hr food record. Subjects were provided with a food record and verbal and written instructions to record the type, quantity and time of all food and beverages consumed. The completed record was reviewed with the subject/parent/guardian by a bionutritionist. Dietary data were entered into the Nutrition Data System for Research (University of Minnesota) for determination of calcium intake. The study was approved by the URMC Institutional Review Board; participants provided written informed consent and assent, as appropriate. Investigators interested in viewing aggregate data or data collection instruments may contact the corresponding author.

## STATISTICAL ANALYSIS

All analysis were performed using STATA 16 (College Station, TX, USA). Standard descriptive statistics were used to summarize participant characteristics. All variables of interest were assessed for normality using the “sktest” function in STATA (11) and by direct visual inspection, prior to analysis. Group differences were assessed *via* two sample t-test (parametric data) or Wilcoxon rank sum (non-parametric data); correlations by Pearson’s (parametric data) r or Spearman’s rho (non-parametric data); and proportions by chi-square test. Linear regression of calcium intake (dependent variable) with tests for interaction were used to assess for group differences (i.e. subject-level demographic and T1D characteristics as independent variables) in associations. Criterion validity was examined by assessing the correlation between calcium intake by FFQ and 24-hour diet record, in a subset of participants (n=96, 40%) who completed both measures. Recruitment goal was at least 200 participants which was predicted *a priori* to provide >80% power to detect correlations ≥ 0.2 at alpha = 0.05.

## RESULTS

**FIGURE 1 ∣:**
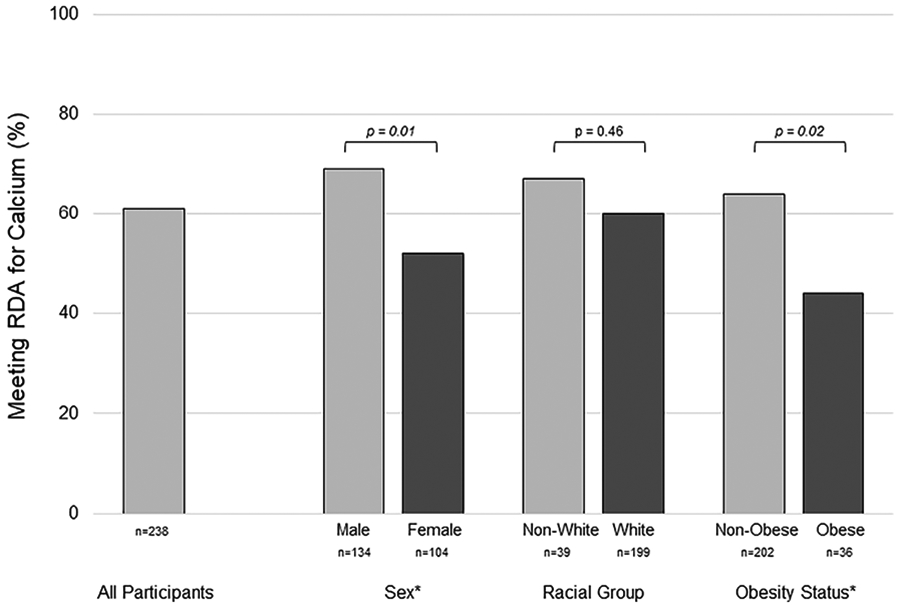
Group differences in the percentage of youth with type 1 diabetes meeting the recommended dietary allowance for calcium assessed by food frequency questionnaire. Inadequate calcium intake was more prevelant in females *versus* males, and in obese *versus* non-obese participants. RDA, recommended dietary allowance * represents statistical significance, p < 0.05, by chi-square test.

Calcium intake was assessed in 238 participants who met eligibility requirements and agreed to participate in the study (~30% of the active URMC pediatric T1D population at the time of the study). Sixty-nine potential participants declined to participate, the most common reason listed was “too busy”. Individuals who declined participation did not differ from enrolled participants by sex or racial group; but were more likely to be under 18 (91% vs. 72%, p=0.01). Characteristics of the study cohort are summarized in Table 1. The median calcium intake (food + supplements) was 1393 mg/d [interquartile range (IQR): 1061-1936]. Median calcium intake was lower in females [1298 mg/d (IQR:954-1676), n=104] than males [1434 mg/d (IQR:1163-2075), n=134], p=0.005, and in obese [1238 mg/d (IQR:1060-1968), n=36] *versus* non-obese [1425 mg/d (IQR:1079-1807), n=202], p=0.02. Calcium intake was below the RDA in 39% of participants. A greater proportion of females (48%) than males (31%), p=0.01, and obese (64%) *versus* non-obese (36%), p=0.02, did not meet the RDA, Figure 1.

The median calcium intake was higher in the 32 participants (14%) reporting use of a calcium supplement [1747 mg/d (IQR: 1264-2397) *vs*. 1371 mg/d (IQR: 1011-1814), p=0.01) *vs*. those who did not; as was the proportion meeting the RDA for calcium intake (78% *vs*. 59%, p=0.04). Eighty-three subjects (35%) reported taking a vitamin D supplement, with median dose of 600 (IQR: 600-1700) international units [15 (IQR:15-43) mcg]. There were no differences between either median calcium intake or proportion meeting RDA for calcium between participants reporting vitamin D or multi-vitamin use, compared to those who did not. The proportion of participants reporting a history of fracture did not differ by calcium or vitamin D supplement use.

**FIGURE 2 ∣:**
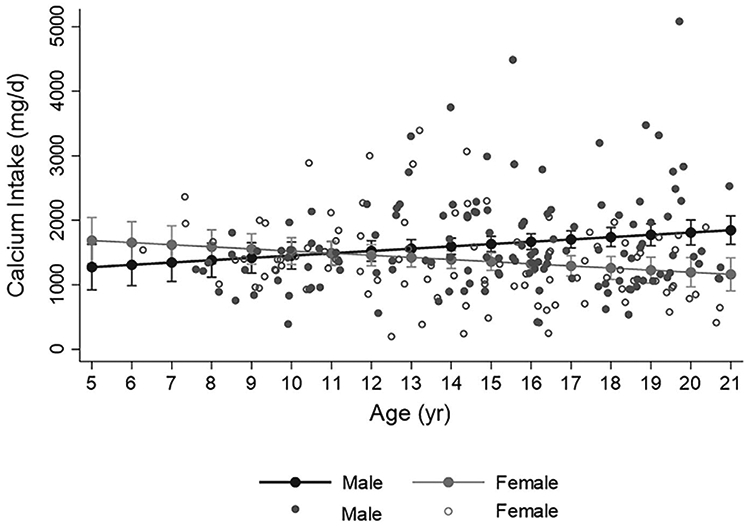
Distribution of calcium intake assessed by food frequency over age in males (closed circles) and females (open circles). Fitted line illustrates the significant sex by age interaction whereby calcium intake increases with age in males and decreases with age in females (p for interaction =0.002, from simple linear regression analyis with calcium intake as dependent variable). Filled circles represent predicted calcium intake (with 95% confidence intervals) for earch year of age.

The relationship between calcium intake and age differed by sex. In males, increasing age was significantly associated with greater intake; whereas in females, the opposite was true, Figure 2, p for interaction = 0.002. For males, the percentage of participants meeting the RDA for calcium was lowest in the less than 8-year old category (38%) and highest in the over 18-year old category (96%), p<0.001. In females, the percentage meeting RDA was highest in the less than 8-year-old category (86%) and lowest in the 14-to-18-year old category (43%), p=0.04. Calcium intake did not differ by racial-ethnic group, and was not associated with HbA1c, average HbA1c from diagnosis, T1D duration, insulin regimen, or fracture history.

Calcium intake by FFQ and 24-hr diet record were significantly correlated in the subset completing both assessments (ρ=0.45, p<0.001, n=96). Calcium intake by FFQ [1383 mg/d (IQR: 1041-1625)] was greater than that by diet record [1146 mg/d (IQR:815-1587], p=0.02 and non-significantly greater in participants assessed in summer [1467 mg/d (IQR:1071-2045)] *versus* school year [1371 mg/d (IQR:1012-1828)], p=0.10.

## DISCUSSION

Optimization of calcium intake is a fundamental tenet of childhood bone health, based upon strong evidence that calcium sufficiency promotes bone mineral accrual and supports the attainment of peak bone mass (12). The positive association between calcium intake and bone mass has also been recently demonstrated in children with T1D (13). Despite the known adverse effects of T1D on bone density and quality (14), few studies have investigated calcium intake in the T1D population. Current recommendations from the American Diabetes Association emphasize the importance of medical nutritional therapy, but do not specifically address calcium (15).

One-third of the participants with T1D in our study did not meet the RDA for calcium. This prevalence was similar to that observed among participants with T1D in the SEARCH study, where 37% (aged <15 years) and 45% (aged >15 years) were below the RDA for calcium (16). Smaller studies conducted outside the United States have reported even higher rates of calcium deficiency (13, 17). The sex- and age-related differences in calcium intake in our study mirrored those reported in a representative sample of US children (18).

Our finding that obesity was a risk factor for deficient calcium intake is of interest given the high prevalence of overweight and obesity among youth with T1D (19). Obese children have been shown to be at risk of micro- and macro-nutrient deficiencies despite excess caloric intake (20). However, the previously reported relationships between calcium intake and obesity have been mixed (21, 22). Emerging data suggest an association between childhood obesity and increased fracture risk (23). These factors support the need for further bone health evaluation in obese children with T1D.

Calcium intake was not significantly associated with HbA1c, insulin regimen, or T1D duration. Concerns have been raised that families of children with T1D may prioritize carbohydrate counting over general healthful eating (24). However, there are limited longitudinal data evaluating the effect of T1D diagnosis on dietary habits. A small study from Finland found that calcium intake decreased following diagnosis (25), but lack of a control group makes it difficult to separate an effect of disease *versus* aging. Our results argue against a T1D disease effect on calcium intake.

This study has limitations. Participants were selected *via* convenience sampling and results may be subject to selection bias. These include factors such as the characteristics of patients attending in-person clinics (i.e. patients with less frequent clinic visits would be less likely to be approached for study participation) and of patients/families who agreed to participate in the study. Simple demographics collected from the electronic health record (EHR) suggested that study participation among those approached for participation may have been influenced by patient age, but not sex or racial/ethnic group. By comparison, when compared to EHR metadata of all current patients with a T1D diagnosis code seen in the URMC Endocrine and Diabetes Clinic, it appeared that participants were less likely to be of Black racial group (5% *vs*. 10%, p =0.01). The sex and average HbA1c of study participants did not differ from the clinic population at large. Generalizability to other academic clinical centers may be affected by the local mix of urban, suburban, and rural patients and population ancestry. FFQs may be subject to recall and information bias related to the use of closed-ended questions, limited items, and inaccurate portion estimations. Steps taken to reduce this bias included modification of the FFQ to include foods consumed by youth and in-person FFQ completion by trained examiners. Calcium intake by FFQ and diet record in this study were significantly correlated at a magnitude similar to previous studies (26). Biochemical parameters including serum calcium and vitamin D concentration are not routinely collected in clinical practice, so no correlations between calcium intake and laboratory markers of bone mineral metabolism can be made in this study sample.

In summary, inadequate dietary calcium intake was common in this cohort of youth with T1D. Female sex, especially during adolescence, and obesity were identified as risk factors for inadequate calcium intake. Strategies to incorporate education regarding calcium intake into the nutritional therapy delivered to youth with T1D should be considered.

## Figures and Tables

**TABLE 1 ∣:** Characteristics of 238 youth with type 1 diabetes assessed for calcium intake.

General	All	Males	Females
**n**	238	134 (56.3)^1^	104 (43.7)
**Age, yrs**	15.2 (11.9-18.4)^2^	15.7 (12.6-18.4)	14.7 (11.1-18.3)
**Racial group**^3^, **n**			
**White**	199 (83.6)	114 (85.1)	85 (81.7)
**Hispanic**	20 (8.4)	11 (8.2)	9 (8.7)
**Black**	12 (5.0)	6 (4.5)	6 (5.8)
**Asian**	3 (1.3)	0 (0.)	3 (2.9)
**Other/missing**	4 (1.7)	3 (2.2)	1 (1.0)
**BMI, kg/m^2^**	22.7 (20.2-25.1)	22.5 (20-24.8)	23.0 (20.4-26.0)
**Obese**^4^, **n**	36 (15.1)	22 (16.4)	14 (13.5)
**Fracture history, n**	56 (23.5)	35 (26.1)	21 (20.2)
**Diabetes**			
**HbA1c, %**	8.2 (7.4-9.3)	8.2 (7.1-9.2)	8.2 (7.5-9.3)
**HbA1c, mmol/mol**	66 (57-78)	66 (54-77)	66 (58-78)
**HbA1c 3-yr average, %**	8.2 (7.6-9.1)	8.2 (7.5-9.1)	8.1 (7.6-8.9)
**HbA1c 3-yr average, mmol/mol**	66 (60-76)	66 (58-76)	65 (60-74)
**T1D duration, yrs**	5.9 (2.8-9.3)	5.9 (2.4-9.3)	5.5 (3.0-9.3)
**Insulin pump use, n**	171 (71.9)	92 (68.7)	79 (76.0)
**Nutritional**			
**Calcium intake, mg/d** ^5^	1393 (1061-1936)	1434 (1163-2075)	1298 (954-1676)*
**Meeting RDA for calcium, n**	146 (61)	92 (68.7)	54 (51.9)*
**Dairy consumption, n**	231 (97)	131 (97.8)	100 (96.2)
**Calcium supplement use, n**	32 (13.5)	14 (10.5)	18 (17.3)
**Vitamin D supplement use, n**	83 (34.9)	46 (34.3)	37 (35.6)
**Multivitamin supplement use, n**	56 (23.6)	32 (23.9)	24 (23.3)

HbA1c, hemoglobin A1c; RDA, recommended dietary allowance; T1D, type 1 diabetes

1 n (%), all such values.

2 median (interquartile range), all such values.

3 Parent/participant reported.

4 BMI >95^th^ percentile (<20 years), >30 kg/m^2^ (≥20 years).

5 Dietary and supplement, from food frequency questionnaire.

* differs significantly from males, p < 0.05.

## Data Availability

The datasets presented in this article are not readily available because this was a single center study of patients with an uncommon disease and therefore participants' identities could potentially be determined for individual level data. Appropriate requests for de-identified data release will be considered in accordance with local IRB policies. Please contact the corresponding authors. Requests to access the datasets should be directed to weberd@chop.edu.
